# Recreational climbers are more conscientious than recreational athletes–a case control study

**DOI:** 10.1186/s13102-022-00483-5

**Published:** 2022-05-25

**Authors:** Gino Steinmetz, Mara Assmann, Jan Hubert, Dominik Saul

**Affiliations:** 1 Department of Occupational and Social Medicine, Georg-August-University of Goettingen, Robert-Koch-Straße 40, 37075 Göttingen, Germany; 2 Department of Trauma, Orthopedics and Reconstructive Surgery, Georg-August-University of Goettingen, 37075 Göttingen, Germany; 3 Division of Orthopaedics, Department of Trauma and Orthopaedic Surgery, University Medical Center Hamburg-Eppendorf, 20521 Hamburg, Germany; 4 Division of Endocrinology, Department of Medicine, Mayo Clinic College of Medicine and Science, Rochester, 55901 MN USA

**Keywords:** Sports psychology, Sports climbing, Climbing psychology, Climbing

## Abstract

**Background:**

Physiological characteristics of climbers have been extensively studied, while the personality of climbers remains poorly understood. Personality research in sports is an established field, predicting long-term success as well as short-term behavior. The goal of our study was to examine recreational climbers’ personality and gain a better understanding of what makes them reach new heights.

**Methods:**

We analyzed a cohort of 50 recreational climbers and 50 non-climbing athletes (control) regarding their personality characteristics. We assessed the BMI, the self-reported climbing grade, and the years of climbing experience. To evaluate the personality of recreational climbers and athletes, we used the German version of the Big Five Inventory (BFI-2), which summarizes the personality in five main categories.

**Results:**

Recreational climbers scored significantly higher in conscientiousness than non-climbing athletes (*p* = 0.04), there was no significant difference between the other four main personality traits. Female climbers scored significantly higher in conscientiousness than male climbers (*p* = 0.02), while female athletes scored higher in openness than male athletes (*p* < 0.01). The climbing group displayed a small but significant negative correlation between conscientiousness and BMI (r = -0.39; *p* < 0.01).

**Conclusion:**

In conclusion, the personality of recreational climbers and recreational athletes differs in conscientiousness, with the climbers showing higher scores. Regarding gender, we found higher levels of conscientiousness in female climbers and higher openness in female athletes in comparison to their male counterparts. A negative correlation between BMI and conscientiousness of the climbers was detected.

## Introduction

After last year’s successful debut at the 2021 Tokyo Olympics, sports climbing is continuing to gain popularity [1, 2]. With this increase in popularity, it can be assumed that the number of recreational athletes will continue to grow. This trend is already observed in an outdoor industry report showing a trend of increased participation in outdoor climbing activities [3]. Another statistic survey done by the Sports & Fitness Industry Association (SFIA) highlights a steady increase in indoor climbing and indoor bouldering participation in the population of the US [4]. Climbing offers a wide variety of subdisciplines, with rock climbing, indoor sports climbing, speed climbing, bouldering and ice climbing being the fundamental ones [1]. Throughout literature, advanced climbers are well studied, but there is still a lack of focus on the recreational climber [1, 5–7]. Mental aspects as well as the relationship between physical and mental aspects of climbing is likewise scarcely covered within the literature. It is well established that in climbing, psychology plays a major role in performance, however there is a strong disproportion between research focusing on the physical against the mental aspect of sports climbing [1]. Only the risk-taking behavior in high-risk sports is a well-researched topic [8, 9]. Llewellyn et al. provide a focused look at the correlation between self-efficacy and risk taking in recreational sports climbers [10]. The authors found that climbers, even recreational ones, high in self-efficacy engaged more frequent in medium and high-risk forms of rock climbing, although the underlying mechanisms remained unclear.

Various experimental designs are used to examine personality in sports. One widely used is the comparison of high-level sportsmen versus low-level sportsmen [11]. Current research suggests that high performance athletes express a lower level of neuroticism [12]. A recent crucial finding is that long-term success in sports, as well as short-term behavior can be predicted by personality traits [13]. In summary, the conjunction of sports und personality continues to arouse interest, however specific research for sports climbing and personality in the recreational climber has not been conducted extensively so far. The climber’s personality in general is largely studied on at least advanced climbers: The advanced climber is described as being high in vigor and mental endurance as well as low in tension, depression, anger, confusion and mood disturbance [1, 6], which is also known as the “Iceberg”-profile [14]. While there is ample research displaying the differences in personality traits between genders, there is hardly any description of the gender differences in recreational athletes [15, 16].

With the current lack of focus on recreational climbers as well as gender differences, the aim of this study was to identify personality traits associated with recreational climbers using the Big Five factor model.

## Materials and methods

### Study setting and design

We prospectively analyzed two cohorts, 50 recreational climbers and 50 non-climbing athletes (40% fitness, 20% running, 18% ball sports, 22% others) regarding their personality traits. In both groups we assessed age, gender and body mass index (BMI). In the climbing group, the highest climbing grade (International Union of Alpine Associations UIAA [UIAA] and International Rock Climbing Research Association [IRCRA]), frequency of training as well as climbing experience in years were assessed. The physical characteristics of the two cohorts were previously published by Assmann et al. [17].

### Sample size determination

For a power of 0.70 (estimated large effect size ρ = 0.3 according to Funder and Ozer [18], α = 0.05), the necessary sample size was calculated to be n = 49 with G*Power (3.1.9.7, University of Duesseldorf, Germany, Faul et al. [19]). Since we accounted for loss, we analyzed 50 climbing and 50 non-climbing athletes.

### Instruments

To evaluate the personality traits we used the German short-form of the Big Five Inventory (BFI-2) [20–22]. The BFI-2 is internally consistent and has been validated in the German language [20].

The BFI-2 consists of 60 items that can be summarized into the five main items: Openness, Conscientiousness, Extraversion, Agreeableness and Neuroticism. The mean Cronbach’s alpha reliability scores for the five summarized traits were high: Openness = 0.84, Conscientiousness = 0.87, Extraversion = 0.86, Agreeableness = 0.81 and Neuroticism = 0.88 [20].

### Data collection procedure

We collected the data, after informed consent, at the RoXx-climbing hall, Goettingen, Germany, as well as the DAV climbing hall Hildesheim, Germany. The data collection took place between 09/18 and 12/19 and each participant needed approximately 45 min for the questionnaire.

For the climbing group, only athletes were considered who regularly climb at indoor climbing centers in Germany with an intermediate and advanced climbing level according to Draper et al. [23]. For the non-climbing group, the athletes performed sport on a regular basis, but no climbing-associated sport like (outdoor) climbing, bouldering or ice climbing.

We excluded athletes under 18 years or subjects with recent surgeries or any acute injuries.

### Study variables

As independent study variables we identified gender, BMI, age, years of climbing experience and the self-reported climbing grade (IRCRA). The dependant variables were Extraversion, Agreeableness, Conscientiousness, Neuroticism and Openness.

### Statistical analysis

Analyses were performed with GraphPad Prism 9.1.2 (GraphPad Software, Inc., San Diego, CA, USA) and SPSS Statistics 26.0.0.0 (IBM, Armonk, NY, USA).

After verification of normal distribution with the d'Agostino Pearson-Test, an unpaired t-test was used if not stated differently. Otherwise, Mann–Whitney test was used. Categorial variables were analyzed with the Fisher’s exact test. Intraclass correlation was used to determine reliability for test–retest. For correlations we used the non-parametric Spearman test. If not stated differently, ± standard deviation is quoted. Depicted values are mean and the 95% confidence interval (CI). Significant differences are marked with asterisks (****p < 0.0001, ***p < 0.001, **p < 0.01, *p < 0.05).

## Results

### Characteristics of climbers and non-climbers

The mean age of the climbing group (30.3 ± 1.8 years) did not differ significantly from the age of the non-climbing group (26.4 ± 1.3 years) (*p* = 0.08). Combining all participants resulted in a mean age of 28.3 ± 11.8 years. There was no significant difference in BMI between the groups (climbing (22.0 ± 0.3 kg/m^2^), non-climbing (22.9 ± 0.5 kg/m^2^, *p* = 0.06)). The gender in the climbing group as well as in the non-climbing group was predominantly male (68% vs. 62%, respectively), not differing between the groups (Fisher’s exact test, *p* = 0.68). The ethnicity was Caucasian. The self-reported climbing grade for the climbing group varied between International Rock Climbing Research Association (IRCRA) levels 10—23 (mean IRCRA 16.88 ± 4.92, mean French: 6c + /7a) (International Union of Alpine Associations UIAA: VI to X-, mean VIII-/VIII). The climbing group had a mean climbing experience of 6.8 (± 7.3) years (Table 1).

**Table 1:** Summary of characteristics of all participants (n = 100)

	Climbers	Athletes	*p*-value
Age	30.3 ± 1.8 years	26.4 ± 1.3 years	0.08 (n.s.)^a^
Sex	16 f | 34 m	19 f | 31 m	0.68 (n.s.)^b^
BMI	22.0 ± 0.3 kg/m^2^	22.9 ± 0.5 kg/m^2^	0.06 (n.s.)^c^
Self-reported climbing grade (IRCRA)	16.88 ± 4.92		
Climbing expierence	6.8 ± 7.3 years		

^a^Mann–Whitney U test

^b^Fisher's exact test

^c^Student's t-test

### Personality traits of climbers and non-climbing athletes

The BFI-2 was used to assess the personality traits of both groups. Comparing both groups we found that conscientiousness differed significantly between the climbers and the non-climbing athletes (*p* = 0.04) with the climbing group scoring higher. There was no significant difference in the other four main categories (Table 2).

**Table 2:** Big five personality traits in the climbing and athlete group (* p < 0.05)

	Climbers	Athletes	*p*-value
Extraversion	3.44 ± 0.63	3.50 ± 0.60	0.57 (n.s.)^a^
Agreeableness	3.94 ± 0.52	3.90 ± 0.45	0.89 (n.s.)^a^
Conscientiousness	3.67 ± 0.59	3.42 ± 0.57	0.04 (*)^a^
Neuroticism	2.45 ± 0.65	2.50 ± 0.52	0.60 (n.s.)^a^
Openness	3.72 ± 0.65	3.70 ± 0.62	0.86 (n.s.)^a^

^a^Mann-Whitney U test

In both groups we compared the personality attributes between genders. In the climbing group, we assessed a significant difference between male and female climbers in conscientiousness, with the female climbers scoring higher (Table 3).

**Table 3:** Comparison of BFI between male and female climbers (* p < 0.05)

	Male climbers	Female climbers	*p*-value
Extraversion	3.4 ± 0.65	3.53 ± 0.60	0.41 (n.s.)^a^
Agreeableness	3.84 ± 0.53	4.15 ± 0.46	0.09 (n.s.)^a^
Conscientiousness	3.53 ± 0.60	3.97 ± 0.48	0.02 (*)^a^
Neuroticism	2.41 ± 0.65	2.53 ± 0.66	0.49 (n.s.)^a^
Openness	3.74 ± 0.60	3.68 ± 0.76	1.00 (n.s.)^a^

^a^Mann-Whitney U test

In the athlete group, the female participants scored significantly higher in openness than the male athletes (*p* < 0.01), while there was no significant difference in the other personality traits (Table 4). There was a significant difference in openness comparing just the female participants of both groups (p = 0.01) with higher openness in female athletes compared to female climbers.

**Table 4:** Comparison of the personality traits between both genders of the athlete group (**** p < 0.0001)

	Male Athletes	Female Athletes	*p*-value
Extraversion	3.33 ± 0.56	3.48 ± 0.68	0.60 (n.s.)^a^
Agreeableness	3.81 ± 0.52	3.91 ± 0.46	0.78 (n.s.)^a^
Conscientiousness	3.74 ± 0.49	3.77 ± 0.72	0.63 (n.s.)^a^
Neuroticism	2.43 ± 0.72	2.73 ± 0.58	1 (n.s.)^a^
Openness	3.58 ± 0.59	4.28 ± 0.33	< 0.01 (****)^1^

^a^Mann-Whitney U test

### Climbing specific correlation of personality traits

**Fig. 1:**
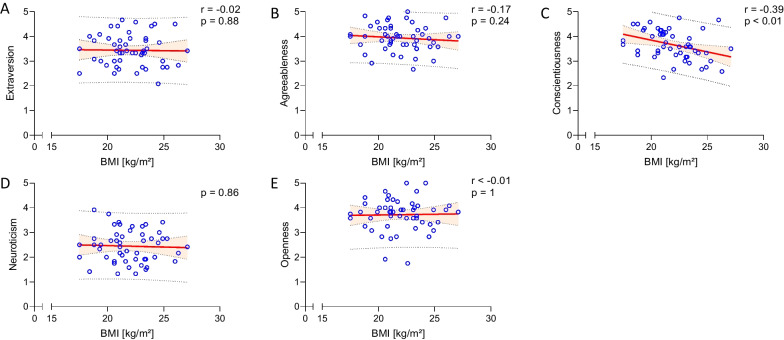
Spearman correlation between personality traits and BMI in the climbing group. We found no correlation between the big five characteristics and BMI in (**a**) extraversion, **b** agreeableness, **d** neuroticism and **e** openness. We did find a significant negative correlation between (**c**) conscientiousness and BMI (r =  − 0.39, *p* < 0.01)

In the climbing group, we used the spearman correlation-test and found a significant negative correlation between conscientiousness and BMI of the participants (Fig. 1). Neither in climbing frequency nor years of experience, we detected a significant correlation to personality traits (data not shown).

## Discussion

This is, to our knowledge, the first study comparing the personality traits of 50 recreational climbers with 50 athletes of other sports as well as providing an in-depth look at gender differences in personality between recreational sportsmen. The characteristics of both groups were comparable to similar studies [24–26]. The aim of this study was to examine the differences in personality between recreational climbers and recreational athletes. A secondary analysis assessed differences between genders of both groups as well as intra-group differences between genders. Recreational climbers scored significantly higher in conscientiousness than matched athletes. We additionally show significant differences in openness between genders of our cohort.

In-depth analyses of climber’s personality are exceedingly rare. Personality traits of athletes however are well described with the Big Five Inventory [24, 27, 28]. In the present study, we found that conscientiousness is significantly higher in recreational climbers compared to regular athletes. We did not find a significant difference in the remaining four personality traits between both groups. This confirmed our initial hypothesis of a prevalent higher conscientiousness in climbing athletes. A possible explanation lies within the nature of climbing as a potentially fatal sport: A higher emphasis on conscientiousness in the participating parties may be lifesaving in certain situations—although an evolutionary advantage of this is not and cannot be proven today. Apart from risking one’s own life, securing your partner on the climbing route is crucial, which may lead to a higher conscientiousness in recreational climbers. In line with that, Malinauskas et al. compared athletes to non-athletes in a similar setup using the NEO Five-Factor inventory (NEO-FFI), which is a commercial tool to assess the five personality dimensions. The NEO-FFI is comparable to the BFI-2 that was used in the present study. Malinauskas et al. showed a significant difference between athletes and non-athletes in conscientiousness [24]. Since all of our participants were recreational athletes of some sport, a direct comparison with this study does not seem feasible. In addition, Malinauskas et al. only recruited male participants. In comparison to athletes, we found that recreational climbers scored significantly higher in conscientiousness. One factor could have been the inclusion of female participants in our study, since female climbers additionally scored higher in conscientiousness than their male counterparts.

In elite alpine climbers ascending Mount Everest, higher levels of extraversion, psychoticism and lower level of neuroticism were found by Egan and Stelmack in comparison to the normative standardization data for males in the 31–40-year age range from the test manual. In recreational climbers, we could not reproduce these findings compared to recreational athletes, while the recreational nature of our participants might be the reason [29]. Burnik et al. compared male Slovenian climbers, with at least one alpinistic expedition to the Himalayas, to male non-athletes who never climbed with the Freiburg Personality Inventory (FPI 114). The FPI 114 is a commercially available questionnaire to assess the personality traits in subjects; it subdivides personality in 9 subdivisions. Burnik et al. did not find differences in extraversion, but found that climbers showed lower levels of neuroticism compared to the control group [25]. Likewise, we did not find a significant difference in extraversion between both groups, but in our cohort no significant difference in neuroticism was found. Burniks study solely focused on male climbers while we also recruited female climbers. This might be one of the reasons for the lack of difference. The fact that the participants of this study were all athletes in some sport might also provide an explanation.

Brandauer found that male climbers had higher depression scales compared to female climbers [30]. Depression is a common mental disorder with a global proportion of 4.4% and is more common in females (5.1%) than males (3.6%) according to estimates of the World Health Organization [31]. Although neuroticism is not linearly linked to depression, common gene variants are presumed since high values can predict later depression [32]. We could not find sex-specific differences in neuroticism, while the base values were low in athletes and climbers. Both groups in our study scored lower than the standardization data in the test manual. The positive effect of climbing and sports on depression is well studied and the low overall values of neuroticism in our groups seem to confirm this effect [33–36]. Additional research by Piepiora et al. shows no significant link between the level of focus and the ability to overcome the fear of falling during a competition. The study reveals that there is a high correlation between climbers’ ability to focus and a high level sports performance [37]. While we were not able to find a correlation between self-reported climbing grade and personality traits, further research might offer insights and demonstrate the differences between recreational and highly advanced climbers. In a similar setup to our study, Marczak and Ginszt compared 30 male and 30 female sports climbers finding that male climbers scored significantly higher in openness as well as agreeableness while there was no significant difference in neuroticism, extraversion and conscientiousness [26]. We encountered opposing results in our study, with the female climbers showing significantly higher conscientiousness and insignificantly higher agreeableness than their male counterparts. One reason might have been the age of 23 ± 1 years in the cohort of Marczak et al., while our cohort was older (30.3 ± 12.7 years) and slightly more experienced (6.8 vs. 6 years of climbing experience). In our athlete group, the female participants also showed significantly higher openness. According to a recent study by Giolla et al., there is a correlation between the gender equality index, in which Germany ranks high, and the sex differences in personalities, showing that a higher gender equality leads to more pronounced differences in personalities [38].

In the climbing group, we found a significant negative correlation between conscientiousness and the BMI of the climbers, which shows that with increasing BMI, the level of conscientiousness of the individuals decreased. While there is currently no research examining the correlation between BMI and personality in climbers, there is ample evidence suggesting BMI does correlate with certain personality types in general. Sutin and Terracciano found in a cross-sectional study with a diverse sample of participants living in the United States (50% female) that high neuroticism was associated with a higher BMI, while conscientiousness was protective of a higher BMI [39]. Comparing these findings with our results, we found a higher conscientiousness correlating with a lower BMI in recreational climbers, although we could not find a correlation between neuroticism and BMI. Our population was, in general, healthy and sports enthusiastic, climbing 2.5 ± 1.2 times a week, which could account for these differences. Gerlach et al. published a review in 2015 where the group categorized conscientiousness and self-control as protective factors towards weight gain [39]. In the climbing group we assessed a BMI of 22.0 ± 0.3 kg/m^2^, which is categorized as a healthy weight, and we also found a negative correlation between BMI and conscientiousness. Gerlach et al. postulates that conscientiousness is associated with self-discipline [39].

Our findings show that recreational climbers score higher in conscientiousness, with female climbers outvaluing their male counterparts. This might point to higher levels of self-discipline in recreational climbers, especially in the female climbers, in comparison to common athletes. Research of personality and the differences between genders in athletes is uncommon. With growing popularity of climbing sports, further research on recreational climbers and gender differences between personalities is warranted.

## Limitations

One limitation of our study is the small sample size. To assess the personality traits of recreational climbers larger scaled studies should be planned.

## Conclusion

In conclusion, the personality of recreational climbers and recreational athletes only differs slightly in conscientiousness, in which the climbing group displays a higher level. We found a lower level of neuroticism in both groups, compared to the standardization data. Examining gender-differences in both groups, we found significantly higher conscientiousness in the female climbers and higher openness in the female athletes in comparison to their male counterparts. We additionally found a negative correlation between BMI and conscientiousness in climbers, which is to be further explored.

## Data Availability

The datasets used and/or analysed during the current study are available from the corresponding author on reasonable request.
