# Correction: Recreational climbers are more conscientious than recreational athletes–a case control study

**DOI:** 10.1186/s13102-023-00638-y

**Published:** 2023-03-08

**Authors:** Gino Steinmetz, Mara Assmann, Jan Hubert, Dominik Saul

**Affiliations:** 1 Department of Occupational and Social Medicine, Georg-August-University of Goettingen, Robert-Koch-Straße 40, 37075 Göttingen, Germany; 2 Department of Trauma, Orthopedics and Reconstructive Surgery, Georg-August-University of Goettingen, 37075 Göttingen, Germany; 3 Division of Orthopaedics, Department of Trauma and Orthopaedic Surgery, University Medical Center Hamburg-Eppendorf, 20521 Hamburg, Germany; 4 Division of Endocrinology, Department of Medicine, Mayo Clinic College of Medicine and Science, Rochester, MN 55901 USA

**Correction to: BMC Sports Science, Medicine and Rehabilitation (2022) 14:94**

https://doi.org/10.1186/s13102-022-00483-5

Following publication of the original article [1], the authors identified an error in the author name of Mara Assmann.

The incorrect author name is: Mara Assman

The correct author name is: Mara Assmann

The author group has been updated above and the original article [1] has been corrected.
